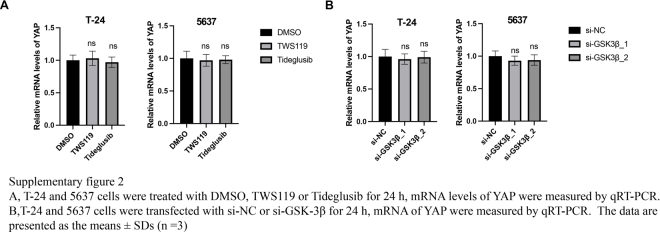# FBXO9 mediated the ubiquitination and degradation of YAP in a GSK-3β-dependent manner

**DOI:** 10.1016/j.jbc.2025.110652

**Published:** 2025-09-01

**Authors:** Yili Jin, Yun Xue, Jiatao Yao, Chengyun Xu, Rui Yu

**Affiliations:** 1 Department of Urology, Affiliated Dongyang Hospital, Wenzhou Medical University, Dongyang, Zhejiang, China; 2 Department of Biochemistry and Molecular Biology, School of Basic Medical Medicine, Health Science Center, Ningbo University, Ningbo, Zhejiang, China; 3 Operating Room, Xinhua Hospital Affiliated to Shanghai Jiao Tong University School of Medicine, Shanghai, China

**Keywords:** YAP, FBXO9, GSK-3β, Hippo signaling pathway, ubiquitination

## Abstract

The Hippo signaling pathway effector YAP (Yes-associated protein) serves as a critical transcriptional regulator involved in a wide range of biological processes, including oncogenesis. Despite its potential as a therapeutic target, pharmacologically targeting the Hippo/YAP axis remains challenging, necessitating further exploration of the mechanisms governing YAP regulation. In this study, we identify the Cullin-RING E3 ligase complex SCF-FBXO9-CRL1 as a novel posttranslational regulator of YAP stability. Mechanistically, FBXO9 recognizes YAP through a conserved degron motif and facilitates its K48-linked polyubiquitination at lysine 76 (K76), thereby promoting proteasomal degradation. Notably, we demonstrate that phosphorylation of YAP at Ser338 and Thr342 by GSK-3β primes YAP for FBXO9 recognition, leading to subsequent ubiquitination. Furthermore, our analysis of the signaling cascade reveals that Akt kinase activity modulates this regulatory axis by influencing the phosphorylation status of GSK-3β. Pharmacological inhibition of Akt signaling leads to YAP degradation in a GSK-3β/FBXO9-dependent manner, significantly enhancing chemosensitivity in cancer models. These findings establish a previously unrecognized regulatory axis involving Akt, GSK-3β, FBXO9, and YAP that controls YAP protein turnover, providing a mechanistic basis for therapeutic strategies that combine Akt inhibitors with conventional chemotherapeutics. Our work advances the understanding of posttranslational YAP regulation and identifies several potential therapeutic targets for YAP-driven malignancies.

## Introduction

Yes-associated protein (YAP) is a key downstream effector of the Hippo signaling pathway. Under normal conditions, YAP collaborates with other transcription factors to regulate a variety of biological processes, including cell proliferation, tissue development, regeneration, and cell death (1). Upon activation, YAP translocate to the nucleus, where it interacts with transcription factors such as the TEA domain family (TEAD) proteins to regulate the expression of various target genes (2). In general, YAP functions as an oncogene, as sustained activation of YAP leads to uncontrolled cell proliferation (3). Furthermore, YAP can upregulate the transcription of antiapoptotic Bcl-2 family members, thereby inhibiting the mitochondrial apoptosis pathway (4). Interestingly, a study showed that peritumoral YAP activation inhibits liver cancer progression in mice (5). The role of YAP in human cancer may be context dependent. Growing evidence indicates that YAP undergoes various post-translational modifications, including SUMOylation, phosphorylation, and ubiquitination (6, 7, 8).

The SCF (SKP1-Cullin1-F-box protein) E3 ubiquitin ligase complex, classified as Cullin-RING ligase 1 (CRL1), represents the largest family of E3 ligases. As the most extensively characterized CRL complex, CRL1 comprises four core components: the adaptor protein SKP1, the scaffold protein Cullin-1 (CUL1), the catalytic RING-box protein 1 (RBX1), and a substrate-specific F-box protein (9). Among these components, the F-box protein serves as the substrate-recognition subunit responsible for target protein engagement, with over 60 distinct F-box proteins currently identified in humans. FBXO9 (F-box protein 9, alternatively designated FBX9 or NY-REN-57) is a 447-amino acid polypeptide characterized by an N-terminal F-box domain that facilitates SKP1 interaction and C-terminal tetratricopeptide repeat (TPR) motifs mediating CUL1 binding (10). FBXO9 exhibits context-dependent dual functionality, demonstrating both oncogenic and tumor-suppressive roles across different malignancies. Notably, upregulated FBXO9 expression has been observed in osteosarcoma clinical specimens, where genomic amplification of chromosome 6p12 harboring FBXO9 correlates with tumor progression (11). Comparative analysis has revealed significantly elevated FBXO9 expression levels in primary multiple myeloma cells derived from patients compared with healthy donor-derived plasma cells. (12). Conversely, FBXO9 downregulation has been documented in acute myeloid leukemia (AML) cohorts, with diminished FBXO9 expression demonstrating significant association with adverse clinical outcomes in this hematological malignancy (13). FBXO9 deficiency is mechanistically linked to aggressive clinicopathological features in ovarian cancer, including advanced tumor staging, histopathological dedifferentiation, and augmented metastatic dissemination. (14). Therefore, FBXO9 might have various biological functions depending on the cellular context.

In the present study, we identify FBXO9 as an E3 ubiquitin ligase targeting Yes-associated protein (YAP) for ubiquitination and proteasomal degradation. Structurally, FBXO9 specifically interacts with YAP through recognition of its canonical PPxY motif. Functionally, this molecular engagement induces K48-linked polyubiquitination cascades that promote YAP destabilization. Mechanistic investigation further identified glycogen synthase kinase 3β (GSK3β) as a critical regulator of this pathway, where GSK3β-mediated phosphorylation establishes the prerequisite molecular context for FBXO9-dependent YAP downregulation. These findings collectively establish the GSK3β/FBXO9 signaling axis as a phosphorylation-regulated ubiquitination mechanism governing YAP protein homeostasis.

## Results

### YAP is a potential substrate of SCF/CRL1-FBXO9

**Figure 1:**
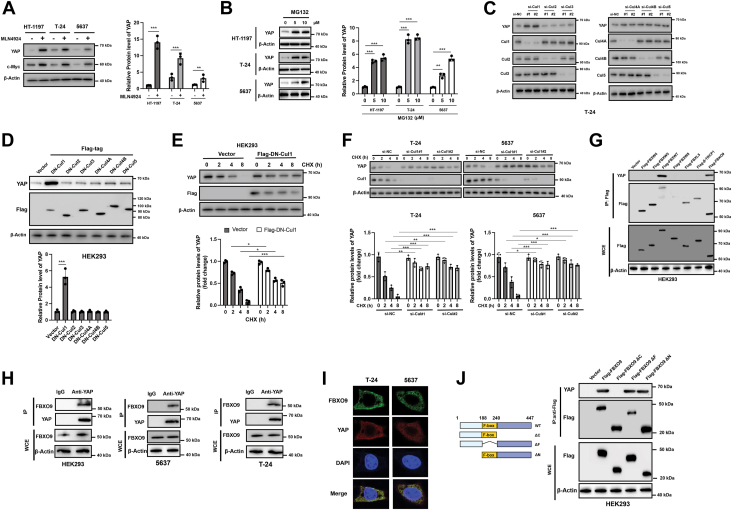
**YAP is a substrate of SCF-FBXO9-Cul1**. *A*, bladder cancer cells (HT-1197, T-24, and 5637) were treated with or without MLN4924 (1 μM) for 24 h, and total cellular proteins were subjected to Western blot analysis with the indicated antibodies (*left*). Quantification analysis of YAP level (*right*). *B*, bladder cancer cells were treated with various doses of MG132 (5 μM, 10 μM) for 24 h, and protein levels were measured *via* western blotting (*left*). Quantification analysis of YAP level (*right*). *C*, T-24 cells were transfected with the indicated siRNAs for 24 h, and total cellular lysates were subjected to western blotting with the indicated antibodies (*left*). Quantification analysis of YAP level (*right*). *D*, HEK293 cells were transfected with empty vector or various Flag-tagged DN (dominant negative) Cullin plasmids for 24 h, and the levels of the indicated proteins were measured *via* western blotting (*Top*). Quantification analysis of YAP levels (*bottom*). *E* and *F*, HEK293 and T-24 cells were transfected as indicated for 24 h and then treated with CHX for various durations. Total protein was subjected to Western blot analysis with the indicated antibodies (*Top*). Quantification analysis of YAP levels (*bottom*). *G*, HEK293 cells were transfected with the indicated vectors, and the resulting cellular lysates were subjected to IP analysis with FLAG-conjugated beads and western blotting. *H*, The endogenous interaction between YAP and FBXO9 was verified by IP analysis. *I*, T-24 and 5637 cells were treated for 6 h with 20 μM MG132 and then fixed, permeabilized, and immunostained. Representative confocal images of cells immunostained for endogenous YAP (*red*) and endogenous FBXO9 (*green*) are shown. Nuclei were counterstained with DAPI (*blue*). *J*, Schematic representation of vectors expressing Flag-tagged wild-type or serial deletion mutants of FBXO9 (*left*). HEK293 cells were transfected with the indicated plasmids for 24 h, after which the resulting cellular lysates were subjected to IP analysis with FLAG-conjugated beads and western blotting (*right*). IP, immunoprecipitation; WCE, whole-cell extracts. The results are representative of three independent experiments. The data was presented as mean ± SD; ∗∗*p* < 0.01; ∗∗∗*p* < 0.001.

**Figure S1:**
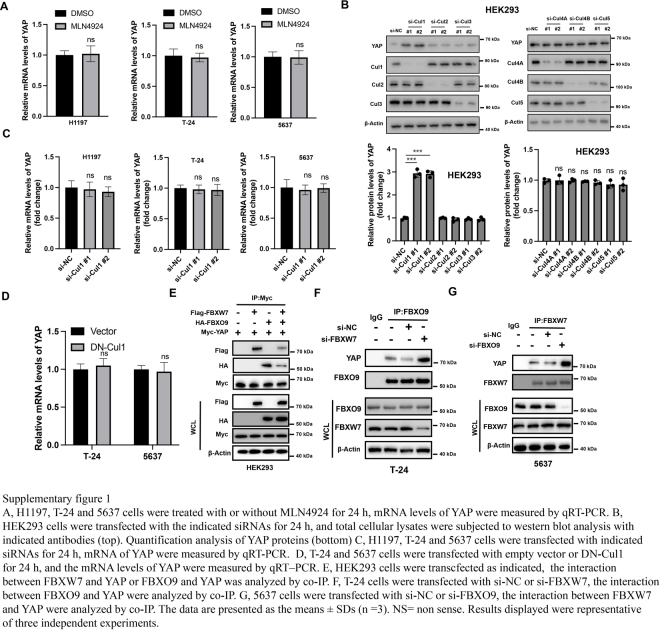


Nearly 20% of proteins can be recognized by Cullin-RING E3 ubiquitin ligases (CRLs) and degraded by the ubiquitin‒proteasome system (UPS) in eukaryotic cells (15). Many transcription factors, such as c-Myc, c-JUN and Notch, are subjected to ubiquitination and degradation mediated by CRLs (16). To investigate whether YAP is also subjected to CRL regulation, MLN4924, a small-molecule inhibitor of CRL, was used. As indicated in Figure 1*A*, YAP is upregulated along with c-Myc, which serves as a positive control in bladder cancer cells (HT-1197, T-24, 5637) after exposure to MLN4924. Moreover, MLN4924 did not affect the mRNA level of YAP (Fig. S1*A*). In addition, treatment with the proteasome inhibitor MG132 also led to the upregulation of YAP in different cells (Fig. 1*B*). Therefore, YAP is likely negatively regulated by CRLs and is degraded by the UPS.

To identify the CRL that is responsible for the degradation of YAP, six different cullins (Culs) were silenced in T-24 and HEK293 cells, and YAP was found to be upregulated in both cell lines after the inhibition of Cul1 (Figs. 1*C*, S1*B*). To further verify this, dominant-negative (DN) Culs were overexpressed in HEK293 cells, and DN-Cul1 markedly induced the accumulation of endogenous YAP protein (Fig. 1*D*). Moreover, the half-life of YAP markedly increased with the overexpression of DN-Cul1 (Fig. 1*E*). Genetic knockdown of Cul1 also increased the half-life of YAP (Fig. 1*F*). However, neither the silencing nor the overexpression of Cul1 affected the mRNA level of YAP (Fig. S1, *C* and *D*). The above findings suggest that SCF/CRL1 is involved in the regulation of YAP, and we next examined which F-box protein, the substrate-binding subunit of SCF, is responsible for the recognition of YAP. Six vectors encoding Flag-tagged F-Box proteins were transfected into HEK293 cells, followed by Flag-bead immunoprecipitation and western blotting for YAP. Interestingly, both FBXW7 and FBXO9 were able to pull down endogenous YAP (Fig. 1*G*). To this end, we investigated the roles of FBXW7 and FBXO9 in binding with YAP. First, we co-expressed FBXW7 and FBXO9 in HEK293 cells. The binding between FBXW7 and YAP was significantly reduced after the overexpression of FBXO9 (Fig. S1*E*). Moreover, the interaction between FBXO9 and YAP was strongly attenuated after the overexpression of FBXW7 (Fig. S1*E*). Next, we silenced FBXW7 or FBXO9 in BC cells. Knocking down FBXW7 promoted the binding of FBXO9 and YAP (Fig. S1*F*). Moreover, inhibition of FBXO9 also increased the interaction between FBXW7 and YAP (Fig. S1*G*). Therefore, FBXO9 and FBXW7 might compete for binding with YAP.

FBXW7 has already been shown to interact with YAP; thus, we focused on the interaction between FBXO9 and YAP (17, 18). We found that endogenous FBXO9 was able to interact with YAP in T-24, 5637, and HEK293 cells (Fig. 1*H*). Immunofluorescence results also confirmed that FBXO9 and YAP were colocalized in T-24 and 5637 cells (Fig. 1*I*). To narrow the region in FBXO9 that interacts with YAP, a series of mutants was created. IP analysis revealed that the C-terminal domain of FBXO9 (aa 240--447) is essential for the binding of FBXO9 to YAP (Fig. 1*J*). Collectively, these observations suggest that YAP can interact with CRL-FBXO9.

### FBXO9 negatively regulates the expression of YAP

**Figure 2:**
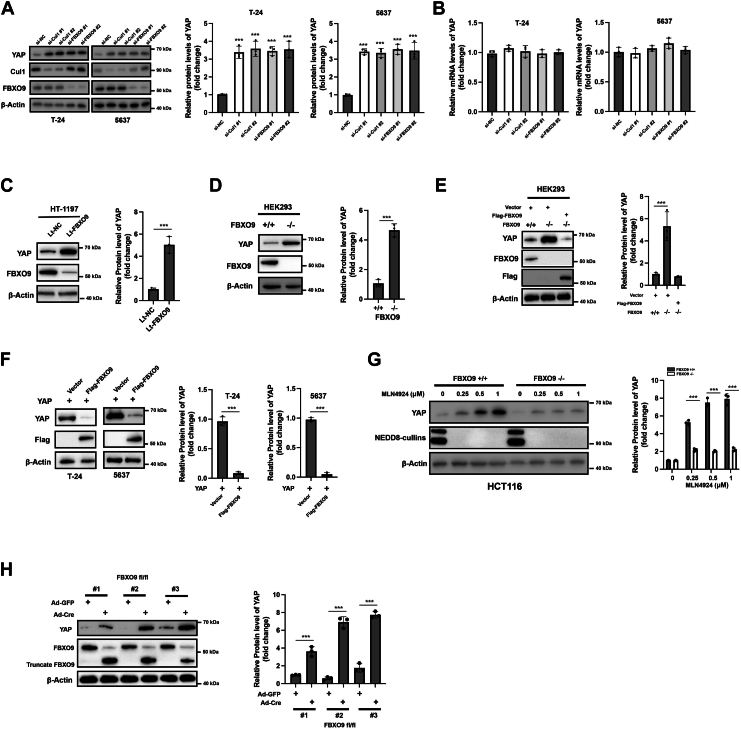
**FBXO9 negatively regulates the protein level of YAP**. *A*, T-24 and 5637 cells were transfected with the indicated siRNAs for 24 h, and protein levels were measured *via* western blotting with specific antibodies (*left*). Quantification analysis of YAP level (*right*). *B*, T-24 and 5637 cells were transfected with the indicated siRNAs for 24 h, and the mRNA levels of YAP were measured by RT-PCR. The error bars indicate the means ± S.D.s, n = 3 from three independent experiments. NS, nonsense. *C*, T-24 cells were infected with Lt-NC or Lt-FBXO9 for 24 h, after which the indicated proteins were measured *via* western blotting (*left*). Quantification analysis of YAP level (*right*). *D*, HEK293 wt and FBXO9 KO cells were lysed, and total cellular lysates were subjected to Western blot analysis with the indicated antibodies (*left*). Quantification analysis of YAP level (*right*). *E*, HEK293 wt and FBXO9 KO cells were transfected with the indicated plasmids for 24 h, and total cellular lysates were subjected to Western blot analysis (*left*). Quantification analysis of YAP level (*right*). *F*, T-24 and 5637 cells were co-transfected with plasmids encoding YAP and Flag-tagged FBXO9 for 24 h, and total cell lysates were subjected to Western blot analysis (*left*). Quantification analysis of YAP level (*right*). *G*, HEK293 wt and FBXO9 KO cells were treated with different doses of MLN4924 for 24 h, and the indicated protein levels were measured *via* western blotting (*left*). Quantification analysis of YAP level (*right*). *H*, FBXO9 ^fl/fl^ MEFs were infected with adenovirus encoding Cre recombinase (Ad-Cre) or Ad-GFP control for 72 h, and the indicated protein levels were measured *via* western blotting (*left*). Quantification analysis of YAP level (*right*). The results are representative of three independent experiments. The data was presented as mean ± SD; ∗∗∗*p* < 0.001.

To further analyze whether YAP is regulated by SCF/CRL1-FBXO9, we examined whether silencing Cul-1 or FBXO9 could affect the levels of YAP. In T-24 and 5637 cells, genetic knockdown of either Cul-1 or FBXO9 resulted in increased YAP protein levels (Fig. 2*A*). However, Cullin-1 or FBXO9 knockdown had little effect on the mRNA level of YAP (Fig. 2*B*). Furthermore, we also observed that lentivirus-mediated knockdown of FBXO9 caused upregulation of YAP in HT-1197 bladder cancer cells (Fig. 2*C*). Higher expression of YAP was also detected in the FBXO9-deleted HEK293 cells than in their wild-type counterparts (Fig. 2*D*). Furthermore, ectopic FBXO9 expression reduced the protein level of YAP in FBXO9−/− HEK293 cells (Fig. 2*E*). The overexpression of FBXO9 also resulted in a reduction in the protein level of YAP in T-24 and 5637 cells (Fig. 2*F*). HCT116 colon cancer cells, which exhibit high expression of Cullin family proteins and sensitivity to MLN4924, were included to validate the universality of FBXO9-mediated YAP regulation across cancer types (19). As shown in Figure 2*G*, MLN4924-induced YAP accumulation was attenuated in FBXO9-deficient HCT116 cells, confirming the conserved role of FBXO9 in CRL1-dependent YAP degradation. Thus, FBXO9 plays an essential role in the degradation of YAP. Finally, we observed that YAP was upregulated in three pairs of primary FBXO9 ^fl/fl^ MEFs (non-cancerous primary cells) infected with adenovirus expressing Cre recombinase (Ad-Cre) to deplete *FBXO9* (Fig. 2*H*). Taken together, these findings highlight the broad relevance of CRL1-FBXO9-dependent YAP regulation.

### FBXO9 shortens the half-life of the YAP protein and promotes its polyubiquitination

**Figure 3:**
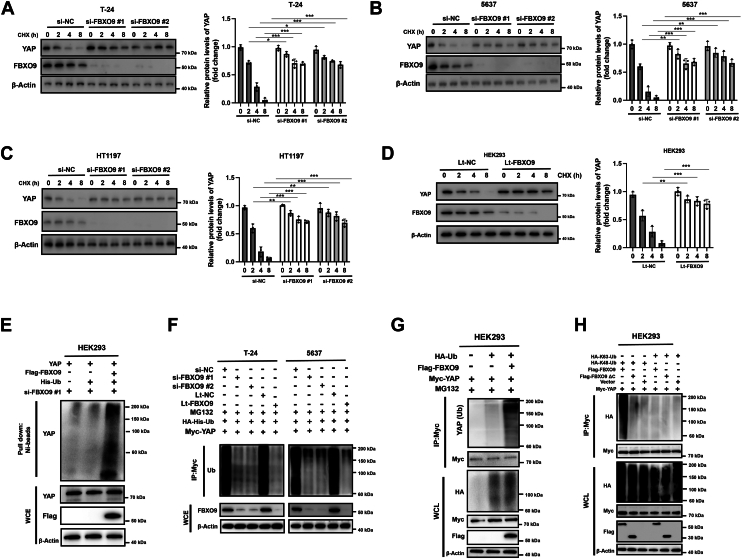
**FBXO9 shortens the YAP protein half-life and promotes the polyubiquitination of YAP**. *A–D*, bladder cancer cells (T-24, 5637, and HT1197) and HEK293 cells were transfected with the indicated siRNAs or lentiviruses for 24 h, after which the cells were treated with CHX for different durations. Total cellular lysates were subjected to Western blot analysis (*left*). Quantification analysis of YAP levels (*right*). The error bars indicate the means ± S.D.s, n = 3 from three independent experiments. *E*, HEK293 cells were firstly transfected with si-FBXO9 for 12 h, then the indicated plasmids were transfected for anther 24 h. Then, the cells were treated with MG132 (20 μM) for 6 h before being harvested. Total cellular lysates were subsequently subjected to His tag pull-down and western blotting analysis. *F*, T-24 and 5637 cells were transfected with indicated siRNAs or Lentivirus for 12 h, then HA-His-UB and Myc-YAP were transected for another 24 h. Cells were treated with MG132 (20 μM) for 6 h before being harvested. Total cellular lysates were IP with Myc and subjected to western blotting analysis. *G* and *H*, HEK293 cells were transfected as indicated for 24 h and then treated with MG132 (20 μM) for 6 h, followed by IP and Western blotting analysis. IP, immunoprecipitation; WCE, whole-cell extracts. The results are representative of three independent experiments.

Since FBXO9 negatively regulates the protein level of YAP, we examined whether FBXO9 affects the half-life of the YAP protein. Three different bladder cancer cell lines and HEK293 cells were transfected with either si-NC/Lenti-NC (negative control) or si-FBXO9/Lenti-FBXO9 to silence FBXO9. The cells were subsequently incubated with cycloheximide (CHX) to block protein synthesis. Silencing FBXO9 significantly prolonged the half-life of YAP in bladder cancer cells (T-24, 5637) and HEK293 cells (Fig. 3, *A*–*D*). Next, we examined whether FBXO9 promoted the degradation of YAP by regulating its polyubiquitination status. An *in vivo* ubiquitination assay revealed that ectopic FBXO9 promoted the ubiquitination of YAP in HEK293 cells (Fig. 3*E*). Moreover, the ubiquitination of YAP was greatly reduced after FBXO9 was knocked down in BC cells (Fig. 3*F*). To further verify that FBXO9 directly regulates the ubiquitination of YAP, Myc-YAP and HA-Ub were cotransfected into HEK293 cells with or without Flag-FBXO9. Immunoprecipitation with anti-Myc beads and Western blot analysis revealed that FBXO9 significantly promoted the ubiquitination of YAP (Fig. 3*G*). Among all ubiquitin chains, those linked *via* K48 and K63 are the most common and well-studied. Polyubiquitylated substrates *via* K48 can be recognized by proteasomes for degradation, whereas K63 linkages usually alter the function of substrates (20). To determine the type of ubiquitin linkage, HEK293 cells were cotransfected with Myc-YAP and Flag-FBXO9 or the truncated mutant Flag-FBXO9 ΔC in the presence of HA-K63-Ub or HA-K48Ub. Immunoprecipitation and Western blot analysis revealed that wild-type FBXO9 promoted the K48-linked ubiquitination of YAP (Fig. 3*H*). Collectively, these observations suggest that FBXO9 functions as an E3 ubiquitin ligase that promotes the ubiquitination of YAP in a binding site-dependent manner in BC cells.

### FBXO9 interacts with a YAP phosphodegron and promotes the polyubiquitination of YAP at the YAP K76 site

**Figure 4:**
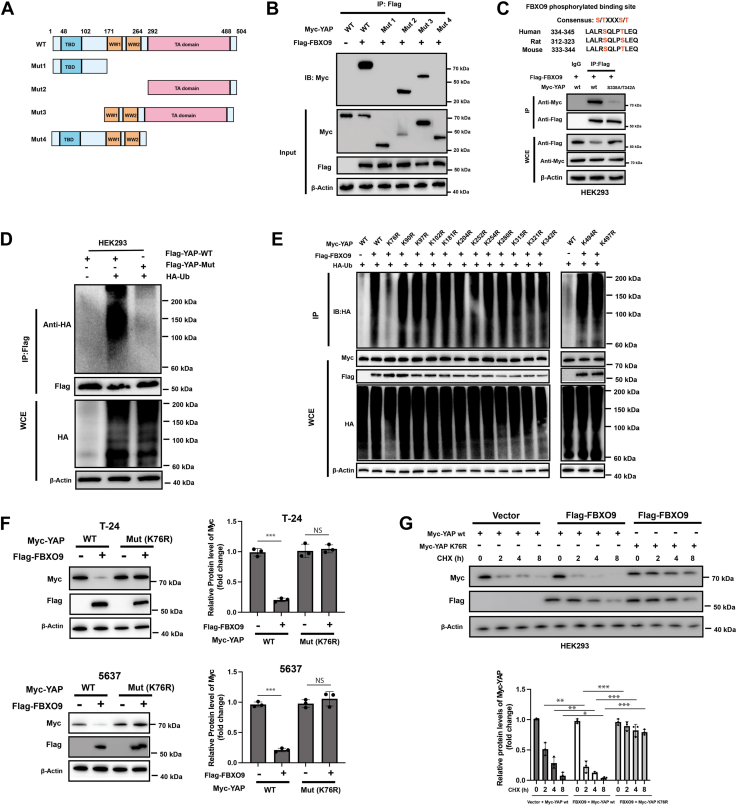
**FBXO9 interacts with YAP *via* the TA domain and promotes ubiquitination at K76**. *A*, schematic diagram of vectors expressing myc-tagged wild-type or serial deletion mutants of YAP. *B*, HEK293 cells were transfected with the indicated vectors for 24 h, and the total cellular lysates were subjected to IP and Western blot analysis. *C*, evolutionarily conserved putative CPD of YAP (*top*). Flag-FBOX9 and Myc-YAP (wild type or S338A/S342A mutant) were co-transfected into HEK293 cells for 24 h, the total cellular lysates were subjected to IP and Western blot analysis. *D*, HEK293 cells were transfected with the indicated vectors for 24 h and then treated with MG132 (20 μM) for 6 h, followed by IP and western blotting analysis. *E*, HEK293 cells were transfected with the indicated vectors for 24 h and then treated with MG132 (20 μM) for 6 h. Total cellular lysates were subjected to IP and Western blot analysis. *F*, T-24 and 5637 cells were transfected as indicated for 24 h, and total cellular lysates were subjected to Western blot analysis (*left*). Quantification analysis of YAP level (*right*). *G*, HEK293 cells were transfected as indicated for 24 h, after which the cells were treated with CHX for different durations. Total cellular lysates were subjected to Western blot analysis (*top*). Quantification analysis of YAP levels (*bottom*).The results are representative of three independent experiments. The data was presented as mean ± SD; ∗∗∗*p* < 0.001.

To further elucidate the interactions between YAP and FBXO9, several subclone variants of YAP were generated (Fig. 4*A*). The IP results revealed that the TA domain of YAP was required for its interaction with FBXO9 (Fig. 4*B*). FBXO9 substrates are often phosphorylated at the phosphodegron motif (CPD), which is required for FBXO9 binding and subsequent ubiquitination by SCF-FBXO9 E3 for degradation (10). Thus, we searched the YAP protein sequence and identified a typical CPD (^338^SQLPT^342^) in the TA domain (Fig. 4*C*). Next, we investigated whether the putative CPD motif is responsible for the regulation of YAP turnover by FBXO9. We substituted S338 and T342 with an alanine to abrogate phosphorylation. As indicated, the S338A/T342A mutation significantly inhibited the interaction between YAP and FBXO9 (Fig. 4*C*). Furthermore, endogenous FBXO9 promoted the polyubiquitination of wild type, but not mutant, YAP (Fig. 4*D*). Next, we investigated the exact sites at which FBXO9 ubiquitinates YAP. Given that the YAP protein has 14 lysine sites for ubiquitin attachment, we generated 14 mutants of YAP. Ubiquitination assays revealed that FBXO9 promoted YAP polyubiquitination mainly at K76 (Fig. 4*E*). Moreover, the YAP K76R mutant was insensitive to FBXO9-mediated degradation in BC cells (Fig. 4*F*). Compared with the wild-type protein, the K76R mutant also prolonged the half-life of the YAP protein (Fig. 4*G*). Collectively, these findings indicate that FBXO9 interacts with YAP through a phosphodegron and promotes the K48-linked polyubiquitination of YAP at the YAP K76 site.

### Activation of GSK-3β is required for the ubiquitination and degradation of YAP

**Figure 5:**
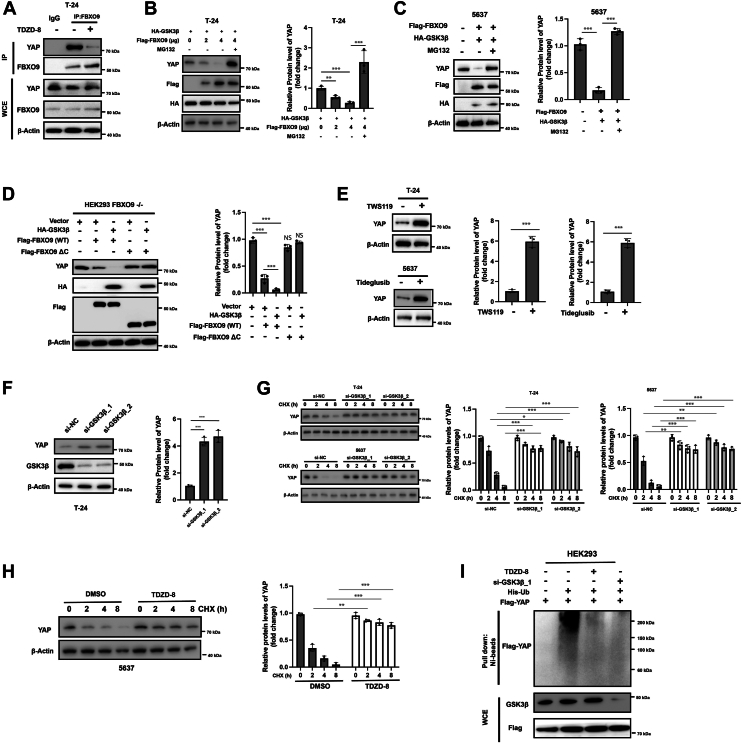
**GSK-3β is required for the CPD and degradation of YAP**. *A*, T-24 cells were pretreated with or without the GSK-3β inhibitor TDZD-8 for 8 h, after which the resulting cellular lysates were subjected to IP and Western blot analysis. *B and C*, T-24 or 5637 cells were transfected as indicated for 24 h, and then the cells were treated with or without MG132 (20 μM) for 6 h. Total cellular lysates were subjected to Western blot analysis with the indicated antibodies (*left*). Quantification analysis of YAP level (*right*). *D*, HEK293 FBXO9-KO cells were transfected with the indicated vectors for 24 h, and the levels of the indicated proteins were measured *via* western blotting (*left*). Quantification analysis of YAP level (*right*). *E*, T-24 and 5637 cells were treated with TWS119 or tideglusib for 24 h, and the indicated protein levels were measured *via* western blotting (*left*). Quantification analysis of YAP level (*right*). *F*, T-24 cells were transfected with the indicated siRNAs for 24 h, and total cellular lysates were subjected to Western blot analysis (*left*). Quantification analysis of YAP level (*right*). *G*, T-24 cells were transfected with the indicated siRNAs for 24 h and then treated with CHX for different durations. Total cell lysates were subjected to Western blot analysis (*left*). Quantification analysis of YAP levels (*right*). *H*, 5637 cells were treated with or without TDZD-8 for 24 h and then exposed to CHX for different durations. Total cell lysates were subjected to Western blot analysis (*left*). Quantification analysis of YAP levels (*right*). *I*, HEK293 cells were transfected as indicated. Then, the cells were treated with MG132 with or without TDZD-8 for 6 h, and the resulting cellular lysates were subjected to His tag pull-down analysis. The results are representative of three independent experiments. The data was presented as mean ± SD; ∗*p* < 0.05; ∗∗∗*p* < 0.001.

F-box proteins are known to recognize phosphorylated substrates (21). The CPD of YAP is essential for FBXO9-mediated YAP degradation, and the CPD has a consensus GSK-3β substrate motif (pS/T-XXX-pS/T) (22). Therefore, we examined whether GSK-3β is involved in the degradation of YAP. The GSK-3β-specific inhibitor TDZD-8 substantially reduced the interaction between FBXO9 and YAP (Fig. 5*A*). HEK293 cells were cotransfected with HA-GSK-3β and Flag-FBXO9, and the YAP protein levels were reduced in a dose-dependent manner (Fig. 5*B*). This effect was fully blocked by the proteasome inhibitor MG132 (Fig. 5, *B* and *C*). Moreover, GSK-3β promoted the degradation of endogenous YAP in the presence of wild-type FBXO9 but not the truncated inactive FBXO9 mutant (FBXO9 ΔC) (Fig. 5*D*). We also observed that treatment with the GSK-3β-specific inhibitor TWS119 and tideglusib markedly increased the protein levels of YAP in T-24 and 5637 cells (Fig. 5*E*). To further analyze the role of GSK-3β in regulating the protein levels of YAP, two siRNAs against GSK-3β were used to knock down GSK-3β (Fig. 5*F*). Consistently, genetic knockdown of GSK-3β also resulted in the upregulation of YAP and extension of the YAP protein half-life (Fig. 5*G*). Moreover, treatment with the GSK-3β inhibitor TDZD-8 also significantly prolonged the protein half-life of YAP (Fig. 5*H*). To this end, genetic or pharmacological inhibition of GSK-3β substantially reduced the polyubiquitination of YAP (Fig. 5*I*). Collectively, our results suggest that GSK-3β is essential for the interaction between YAP and FBXO9 and the subsequent degradation of YAP.

### GSK-3β directly phosphorylates YAP

**Figure 6:**
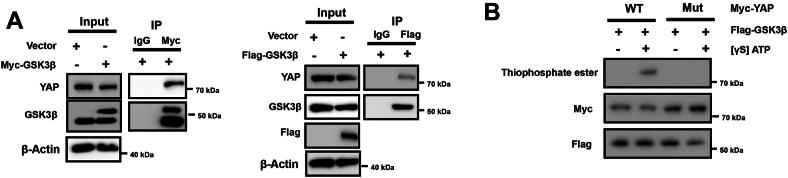
**GSK-3β directly interacts with YAP and phosphorylates it**. *A*, HEK293 cells were transfected as indicated for 24 h, and IP was conducted for GSK-3β with anti-Myc or anti-Flag antibodies, followed by Western blot analysis. *B*, Phosphorylation of purified GST-tagged YAP wt and YAP mut by GSK-3β was conducted by mixing 1 mM [γ^32^P] ATP with kinase buffer and incubating at 37 °C for 30 min. The protein mixtures were separated by 10% SDS‒PAGE and subjected to Western blot analysis. The results are representative of three independent experiments.

Next, we investigated whether GSK-3β interacts with YAP. IP assays revealed that when Flag-GSK-3β or Myc-GSK-3β was pulled down, there was an interaction between GSK-3β and YAP (Fig. 6*A*). Since the CPD of YAP is essential for FBXO9-mediated YAP degradation, we asked whether this site is phosphorylated by GSK-3β. To this end, an *in vitro* kinase assay was conducted to examine whether GSK-3β directly phosphorylates YAP. We cloned and overexpressed the wild-type YAP (YAP wt) and the mutant form (YAP mut) which contains S338A/T342A mutations. As indicated in Figure 6*B*, GSK-3β could phosphorylate the YAP wt protein, but the YAP mut protein was not phosphorylated, and β-catenin was used as a positive control. Therefore, GSK-3β can directly phosphorylate YAP.

### Stress conditions promote the ubiquitination and degradation of YAP

**Figure 7:**
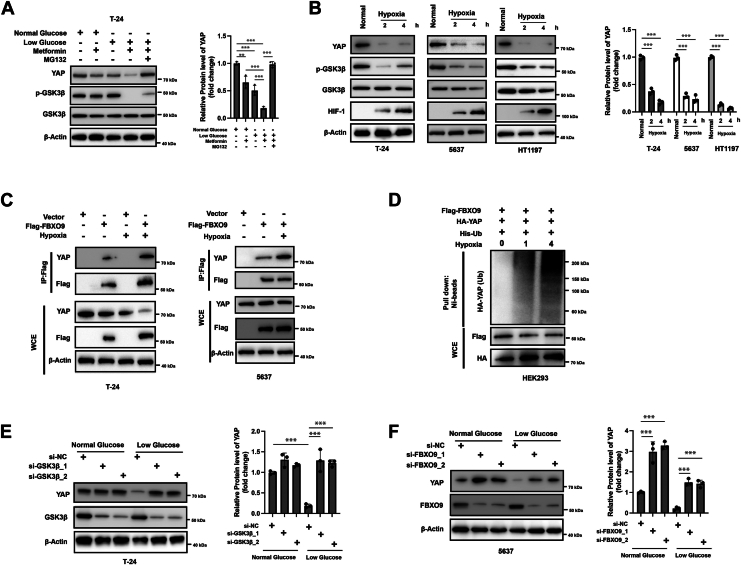
**Stress conditions negatively regulate the expression of YAP**. *A*, T-24 cells were cultured in normal glucose medium (10 mM) or low glucose medium (1 mM) and replenished every 4 h with or without metformin (5 mM). Then, the cells were exposed to MG132 or not for another 6 h. Total cellular lysates were subjected to Western blotting analysis (*left*). Quantification analysis of YAP level (*right*). *B*, T-24, 5637 and HT1197 cells were cultured as described, and the indicated proteins were analyzed by western blotting (*left*). Quantification analysis of YAP level (*right*). *C*, T-24 and 5637 cells were transfected as indicated for 12 h and then cultured under normal or hypoxic conditions for another 4 h. IP with an anti-Flag antibody and western blotting were subsequently conducted. IP with an anti-Flag antibody and western blotting were subsequently conducted. *D*, HEK293 cells were transfected as indicated, treated with MG132 for 6 h and subjected to hypoxic conditions for one or 4 h, followed by a ubiquitylation assay. *E*, T-24 cells were transfected with siRNAs against GSK-3β or negative control (NC), and the cells were cultured under normal or low-glucose conditions for 24 h. Total cellular lysates were subjected to Western blot analysis with the indicated antibodies (*left*). Quantification analysis of YAP level (*right*). *F*, 5637 cells were transfected with siRNAs against YAP or negative control (NC), and the cells were cultured under normal or low-glucose conditions for 24 h. Total cellular lysates were subjected to Western blot analysis with the indicated antibodies (*left*). Quantification analysis of YAP level (*right*). The results are representative of three independent experiments. The data was presented as mean ± SD; ∗∗∗*p* < 0.001.

A previous study indicated that stress conditions can activate the GSK-3β signaling pathway to regulate its downstream targets (23). Therefore, we examined whether stress conditions could also regulate the GSK-3β/FBXO9/YAP axis. It has been reported that a combination of low glucose and metformin can activate GSK-3β (24). The same stress conditions were applied, and combined treatment with low glucose and metformin-activated GSK-3β and substantially reduced the endogenous level of YAP (Fig. 7*A*). Moreover, this effect was fully blocked by MG132 treatment (Fig. 7*A*). Moreover, short-term exposure of cells to hypoxia (1% O_2_) resulted in the downregulation of YAP (Fig. 7*B*). Moreover, hypoxia promoted the interaction between YAP and FBXO9 (Fig. 7*C*) and the ubiquitination of YAP (Fig. 7*D*). Next, we investigated the role of GSK-3β and FBXO9 in the degradation of YAP caused by low glucose. Interestingly, silencing of GSK-3β fully restored the protein levels of YAP under low-glucose conditions (Fig. 7*E*). Moreover, FBXO9 knockdown only partially rescued the protein level of YAP under low-glucose conditions (Fig. 7*F*). Taken together, these findings indicate that YAP levels are regulated by stress conditions and that both GSK-3β and FBXO9 are involved in the degradation of YAP caused by low glucose.

### Inhibition of Akt signaling sensitizes bladder cancer cells to gemcitabine by targeting YAP through FBXO9

**Figure 8:**
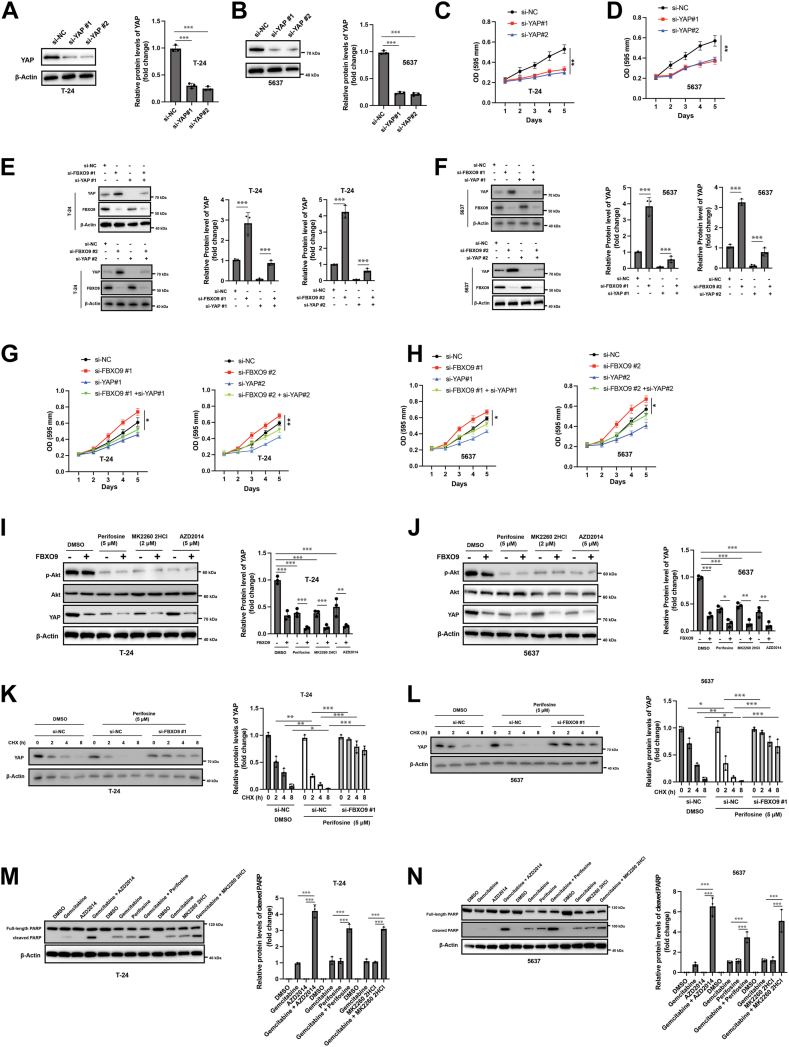
**The Akt/GSK-3β/FBXO9/YAP axis regulates the proliferation and chemosensitivity of bladder cancer cells**. *A–B*, T-24 (*A*) and 5637 (*B*) cells were transfected with the indicated siRNAs for 24 h, and total protein was subjected to Western blot analysis (*left*). Quantification analysis of YAP level (*right*). *C* and *D*, T-24 (*C*) and 5637 (*D*) cells were transfected as described, and cell viability was measured by a CCK-8 assay. *E* and *F*, T-24 (*E*) and 5637 (*F*) cells were transfected with the indicated siRNAs for 24 h, and total protein was subjected to Western blot analysis (*left*). Quantification analysis of YAP level (*right*). *G* and *H*, T-24 (*G*) and 5637 cells were transfected as indicated, cellular viability was measured at different time. The data are presented as the means ± SDs (n = 3), ∗*p* < 0.05. *I* and *J*, T-24 (*I*) and 5637 (*J*) cells were treated with the indicated agents for 24 h, and total cellular lysates were subjected to Western blotting with the indicated antibodies (*left*). Quantification analysis of YAP level (*right*). *K* and *L*, T-24 (*K*) and 5637 (*L*) cells were transfected with the indicated siRNAs for 24 h, and then the cells were treated with 1 μM perifosine for another 24 h. YAP protein turnover was examined 2 h after inhibitor treatment *via* a cycloheximide chase assay (*left*). Quantification analysis of YAP level (*right*). *M* and *N*, T-24 (*M*) and 5637 (*N*) cells were treated as indicated for 24 h, and the cleavage of PARP was examined by western blotting (*left*). Quantification analysis of cleaved PARP level (*right*). The results are representative of three independent experiments. The data were presented as mean ± SD; ∗*p* < 0.05; ∗∗*p* < 0.01; ∗∗∗*p* < 0.001.

Finally, we examined the biological effects of FBXO9-mediated downregulation of YAP. Previous studies have indicated that YAP acts as an oncogene and promotes the growth of bladder cancer cells (25, 26). To this end, we silenced YAP in T-24 and 5637 cells (Fig. 8, *A* and *B*). Cellular viability assays revealed that knockdown of YAP markedly reduced the viability of both cell lines (Fig. 8, *C* and *D*). To further verify the role of the FBXO9/YAP axis, we also silenced FBXO9 alone or in combination with YAP downregulation in T-24 and 5637 cells (Fig. 8, *E* and *F*). The downregulation of FBXO9 promoted the viability of bladder cancer cells, which was substantially attenuated by the knockdown of YAP (Fig. 8, *G* and *H*). Thus, these findings suggest that FBXO9 and YAP coordinately regulate the proliferation of bladder cancer cells. Numerous studies suggest that PI3K/Akt signaling antagonizes GSK-3β; hence, we speculated that hyperactivation of the PI3K/Akt pathway could stabilize the YAP protein. To this end, we examined whether PI3K inhibitors could sensitize bladder cancer cells to gemcitabine treatment by targeting YAP for degradation *via* GSK-3β/FBXO9. Three PI3K pharmacological inhibitors (perofosine, AZD2014 and MK2260 2HCl) were applied, and the Western blotting results revealed that these inhibitors successfully inhibited Akt signaling (Fig. 8, *I* and *J*). Notably, the expression of FBXO9 in combination with PI3K inhibitors resulted in the downregulation of YAP (Fig. 8, *I* and *J*). Furthermore, cycloheximide chase experiments revealed enhanced turnover of the YAP protein in response to Akt inhibitors, and silencing FBXO9 completely abolished this effect (Fig. 8, *K* and *L*). Western blotting analysis also revealed that combined treatment with PI3K inhibitors and gemcitabine led to increased apoptosis, as evidenced by cleaved PARP (Fig. 8, *M* and *N*). Collectively, these findings suggest that Akt signaling acts upstream of the GSK-3β/FBXO9/YAP axis and that inhibition of PI3K signaling sensitizes bladder cancer cells to gemcitabine at least partially *via* the GSK-3β/FBXO9/YAP axis.

## Discussion

As a downstream effector of the Hippo signaling pathway, YAP normally functions as a transcriptional coactivator and cooperates with other transcription factors to promote the development of various cancers (27). Aberrant upregulation of YAP is correlated with tumor progression, tumor metastasis, chemoresistance, and poor prognosis (28). The clinical importance of therapeutic targeting of the Hippo/YAP axis is widely acknowledged, and several Hippo/YAP inhibitors, such as verteporfin, VGLL4, and SuperTDU, have been developed (29). However, these agents have prominent shortcomings, such as cellular toxicity, instability to light exposure, and a low ability to cross the cell membrane (30). Therefore, fully elucidating the mechanisms underlying the regulation of YAP, which might be helpful for developing better strategies to inhibit the Hippo/YAP signaling pathway, is necessary. In the present study, we found that YAP is a new substrate of the SCF-FBXO9 E3 ligase based on the following evidence: (1) MLN4924, a general molecular inhibitor of CRL E3s that inhibits neddylation of all cullins, induces the upregulation of YAP at the protein level but not at the mRNA level; (2) endogenous YAP bound to FBXO9; (3) genetic inhibition/depletion of FBXO9 caused the upregulation of YAP by prolonging its protein half-life; and (4) FBXO9 promotes the polyubiquitination and degradation of YAP. Furthermore, we revealed that GSK3β activation is required for FBXO9-mediated polyubiquitination and degradation of YAP. Thus, our investigation identified YAP as a new substrate of FBXO9 and revealed that it is negatively regulated by FBXO9.

YAP and its paralog transcriptional coactivator with PDZ-binding motif (TAZ) are the major downstream effectors of the Hippo pathway (31). Accumulating evidence suggests that YAP acts as an oncogene in various cancers. For example, sustained activation of YAP causes abnormal proliferation of multiple types of cancer cells, such as breast cancer, liver cancer, colorectal cancer, and bladder cancer cells (32, 33, 34, 35). YAP also confers chemoresistance in lung cancer, osteosarcoma, and squamous cell carcinoma (36, 37, 38). Upregulation of YAP facilitates the escape of tumor cells from contact inhibition and thereby promotes cell survival, proliferation, migration and invasion (32). The activity of YAP can be regulated at different levels. LATS1/2 kinase, a key component of the Hippo cascade, can directly phosphorylate YAP and inhibit the translocation of YAP from the cytoplasm to the nucleus (39). YAP can also be modified at the posttranslational level. For example, the E3 ligase MIB2 promotes the ubiquitination and degradation of YAP in endothelial cells (40). Parkin, another E3 ligase, enhances the ubiquitination and degradation of YAP in various tumor cells (41). Previous investigations have indicated that YAP is also regulated by the F-box proteins FBXW7 and β-TrCP in hepatocellular carcinoma and pancreatic tumors, respectively (17, 18). On the other hand, several deubiquitinating enzymes have been shown to increase the stability of YAP. For example, USP19 was able to decrease the ubiquitination of YAP in hepatocellular carcinoma (42). Moreover, UCHL3 was also identified as a deubiquitinase that can directly interact with YAP and inhibit its degradation (43). To our knowledge, this study is the first to demonstrate that FBXO9 directly interacts with YAP and promotes its ubiquitination and degradation. Notably, our results suggest that FBXO9 and FBXW7 compete for binding with YAP, and further investigations are needed to elucidate the details involved.

Through co-IP assays, we revealed that FBXO9 directly interacted with the TA domain of YAP. The TA domain of YAP exhibits strong transactivation properties, and this domain is also responsible for the binding of other proteins, such as ZO2 and NHERF2 (44). Typically, the presence of a degron in the substrate is required for F-box proteins to bind with, ubiquitinate and degrade the substrate. In the degron, a negative charge resulting from phosphorylation (*i.e.*, p-Ser or p-Thr) or acidic amino acid residues downstream of the +4 position of the central phosphothreonine (p-Thr) often exists (16). This negative charge enables the substrate to directly interact with F-box proteins (15). In our study, we revealed that there are two degrons in YAP and mutation of S338A/T342A significantly abrogated the association between YAP and FBXO9. There are several modes of polyubiquitination, such as the ubiquitination of lysine sites 9, 11, 27, 29, 33, 48, and 63 and linear ubiquitination (45). Among them, K48-linked ubiquitination, which is usually linked to protein degradation, has been the most extensively studied, while other forms of ubiquitination can affect protein location and/or other functionalities (46). We found that FBXO9 could facilitate K48-linked polyubiquitination and degradation of the YAP protein, which is in accordance with the findings of a previous study showing that YAP undergoes K48-linked polyubiquitination and is degraded in esophageal squamous carcinoma cells (47). Notably, YAP can also be polyubiquitylated *via* K63 linkages, which results not only in degradation but also in functional changes in YAP. For example, the E3 ligase SKP1 promoted the K63-linked polyubiquitination of YAP, which promoted YAP nuclear translocation and transcriptional activity (48). Therefore, YAP might be subjected to different forms of polyubiquitination depending on physiological conditions, and further investigations are needed to verify this hypothesis.

Although its role in ubiquitination has been confirmed, FBXO9 is an understudied member of the F-box protein family with limited functional implications. FBXO9 targets a few substrates for ubiquitination-dependent degradation, such as Sox10, p53, DPPA5, and PPARγ (10, 49). We investigated FBXO9 by identifying YAP as a novel substrate in bladder cancer cells. FBXO9 decreased the protein but not the mRNA level of YAP. Moreover, the half-life of YAP was decreased by forced expression of FBXO9, as determined *via* cycloheximide (CHX) analysis. FBXO9 directly interacts with YAP and promotes its ubiquitination and subsequent degradation in a proteasome-dependent manner. Specifically, we revealed that the F-box domain of FBXO9 was critical for binding to YAP. This observation is in accordance with previous studies showing that FBXO9 recognizes its substrates *via* its F-box domain (10, 13, 49). Our findings suggest that FBXO9 functions as a negative regulator of YAP. Notably, a recent study revealed that FBXO9 mediates the degradation of FBXW7, which also acts as an E3 ligase and regulates the ubiquitination and degradation of YAP (50). Therefore, it is reasonable to speculate that FBXO9 might increase the level of YAP under certain circumstances. This may also at least partially explain why FBXO9 functions as an oncogene or tumor suppressor in different types of cancers.

GSK-3β has been implicated as an upstream kinase that regulates the turnover of various substrates targeted by F-box proteins such as FBXW7 and β-TrCP (15). However, there are few reports on the role of GSK-3β in FBXO9-mediated protein turnover. In accordance with a previous report, we applied low glucose in combination with metformin to activate GSK-3β (24). The YAP levels were significantly reduced under such conditions. Moreover, we also found that GSK-3β knockdown/inhibitor treatment significantly reduced the interaction between YAP and FBXO9. Furthermore, the data indicated that the effect of GSK-3β on YAP degradation relies on intact FBXO9. GSK-3β is a highly conserved serine/threonine protein kinase that is widely expressed in different human tissues (51). GSK-3β usually recognizes substrates with p-S/T-XXX-S/T motifs, where the first S/T is the targeted phosphorylation site (22). Here, we identified Ser338 at the C-terminal region of YAP as the GSK-3β phosphorylation site. Both *in vitro* kinase assays and cell-based *in vivo* analyses confirmed that GSK-3β directly phosphorylated Ser338, and this phosphorylation promoted the subsequent ubiquitination and degradation of YAP. However, our study cannot exclude the possibility that other consensus motifs in YAP are also phosphorylated by GSK-3β and/or other kinases.

Another biologically significant finding of our study is that the GSK-3β/FBXO9/YAP regulatory axis is controlled by the PI3K signaling pathway. We previously reported that the PI3K/Akt signaling pathway was activated in bladder cancer cells and might be a potent therapeutic target (52). Multiple PI3K inhibitors are under clinical evaluation and show promising effects alone or in combination with other agents against several cancers, including bladder cancer (53). Our results indicated that inhibition of the PI3K signaling pathway destabilized YAP in bladder cancer cells with functional FBXO9 and therefore sensitized the cells to the chemotherapeutic agent gemcitabine. Consistent with our findings, accumulating evidence suggests that there is a close correlation between PI3K and YAP. For instance, Zhao *et al.* reported that PI3K was able to positively regulate YAP during mammary tumorigenesis (54). However, the underlying mechanisms are still not fully understood. Our findings provide novel insights into this phenomenon and have certain clinical implications. Since resistance to gemcitabine is still an outstanding clinical problem, our results suggest that targeting the PI3K/GSK-3β/FBXO9/YAP axis might be a potent strategy to overcome this resistance.

There are several limitations of our study. First, our investigations were conducted *in vitro*; therefore, it is worthwhile verifying our findings *in vivo*. For example, cell line xenograft and/or patient-derived xenograft (PDX) mouse models were used to verify our observations. Second, it would be worthwhile to test our major observations in clinical samples, such as the correlation between the expression of FBXO9 and YAP. Third, while our study primarily utilized bladder cancer cell lines (T-24, 5637, HT-1197) to model YAP regulation, we validated key findings in HCT116 colon cancer cells and non-cancerous MEFs to ensure broad applicability. Although cell line-specific cofactors (*e.g.*, varying levels of GSK-3β, Akt, or Hippo pathway components) could theoretically influence YAP stability, the consistent results across diverse models suggest that the FBXO9/GSK-3β/YAP axis operates independently of tissue-specific contexts. Future studies in patient-derived models or clinical samples will further validate these observations.

In conclusion, we showed that SCF-Cul1-FBXO9 can directly interact with YAP and promote its ubiquitination and degradation. In addition, GSK-3β kinase is essential for FBXO9-mediated turnover of YAP. Short-term hypoxia treatment decreased the protein level of YAP by enhancing FBXO9–YAP binding and subsequent polyubiquitination of YAP. Finally, PI3K signaling acts upstream of GSK-3β, and inhibition of PI3K leads to overcoming gemcitabine resistance through the FBXO9/YAP axis. Thus, our findings provide novel insight into the regulation of YAP.

## Experimental procedures

### Cell culture and transfections

The T-24, 5637, HCT116, and HT-1197 cell lines were purchased from Pricella Biotechnology. HEK-293 cells were obtained from the Shanghai Bank of Cell Lines, Chinese Academy of Sciences. HEK-293 and FBXO9 KO cells were obtained from Beyotime Biotechnologies. These cell lines were authenticated and cultured in RPMI 1640 medium supplemented with 10% fetal bovine serum (FBS, HyClone) and 1% penicillin/streptomycin (Gibco). Murine embryonic fibroblasts (MEFs) were a generous gift from Prof Weijun Wu (Zhejiang University) and cultured in DMEM supplemented with 15% FBS, 2 mM L-glutamine, and 0.1 mM MEM nonessential amino acids. All cells were cultured in a humidified incubator with 5% CO_2_ at 37 °C. The cells were passaged at 70 to 80% confluence by dissociation from the culture flask using 0.25% trypsin-EDTA (Gibco). The cells were resuscitated every 3 months and tested negative for *mycoplasma* contamination. Lipofectamine 3000 (Life Technologies) was used for plasmid transfection. The T-24, 5637, and HT-1197 bladder cancer cell lines were selected for their relevance to YAP-driven oncogenesis and chemoresistance. HEK-293 cells were used for overexpression and interaction studies due to their high transfection efficiency. HCT116 colon cancer cells were included to validate CRL1-FBXO9 activity in a non-bladder cancer context, while MEFs provided a non-transformed model to exclude cancer-specific artifacts.

### Antibodies, chemical reagents, plasmids, and siRNAs

The following antibodies were obtained from Cellular Signaling Technology: anti-Cul1 (4995), anti-Cul3 (10,450), anti-Cul4A (2699), anti-Cul4B, anti-Cul5, anti-YAP (4912), anti-GSK-3β (9315), anti-phospho-GSK-3β (5558), HIF-1 (3716), anti-Tubulin (2146), anti-Flag (14,793), anti-Myc (5605), anti-Ubiquitin (3936), anti-HA (3724), anti-GST (2622), anti-Akt (9272), anti-phospho-Akt (4060), and anti-PARP (9541). The following antibodies were purchased from Abcam: anti-FBXO9 (ab115521), anti-Cul2 (ab166917), anti-Cul4B (ab317014), anti-Cul5 (ab264284), and anti-NEDD8 (ab194582). The secondary antibodies were purchased from Jackson Laboratory (USA). All the antibodies were diluted according to the manufacturer's instructions.

The following beads were used in the study: c-Myc AC beads (HY-K0206, MedChemExpress), anti-Flag M2 affinity beads (M8823, Sigma‒Aldrich), Pierce Ni-NTA beads (78,605, Thermo Fisher Scientific), and Sepharose G beads (ab193262, Abcam). The following pharmacological agents were obtained from Selleck Chemicals: MLN924 (S7109), cycloheximide (CHX) (S7418), MG132 (S2619), TDZD-8 (S2926), tideglusib (S2823), TWS119 (S1590), gemcitabine (S1714), perifosine (S1037), MK-2206 2HCl (S1078), and AZD2014 (S2783). All other routine chemicals were obtained from Sigma‒Aldrich.

For the plasmids, the coding sequences of FBXW4, FBXW5, FBXW7, FBXW8, FBXL5, β-TrCP1, FBXO9, FBXO11 and Skp2 were subcloned and inserted into pCMV5-Flag *via* a One-Step SuperClone Kit (Hieff BioTech). Flag-tagged DN-Cul1/Cul2/Cul3/Cul4A/Cul4B/Cul5 were designed and generated by FuBiotherapeutic Ltd. The plasmids encoding FBXO9 mutants and YAP mutants were created *via* site-directed mutagenesis with the Flag-tagged FBXO9 vector or Myc-tagged YAP as the template *via* a QuickMutation plus kit (Beyotime Biotechnologies) according to the manufacturer's instructions. HA-ubiquitin, Myc-GSK3β and Flag-GSK3β plasmids were obtained from OBio Technologies (Shanghai, China). To knockdown different targets, siRNAs were designed by GenePharma Ltd (Suzhou, China). The sequences of the siRNA oligos used in this study are listed in Table S1. For lentivirus-based shRNA knockdown, the oligonucleotides were cloned and inserted into the pLKO.1 vector. The lentiviral particles were generated in HEK-293T cells *via* the transfection of the pLKO.1 plasmid along with the packaging plasmid psPAX2 and the envelope plasmid pMD2G. 12 hours after transfection, the medium supplemented with lentiviral particles was collected, filtered through 0.45 μm filters and collected by centrifugation at 4 °C. The generation of lentiviruses was conducted by OBio Technologies.

### Protein ubiquitination assay

HEK293T cells were cotransfected with HA-His-ubiquitin and other relevant constructs. Forty-eight hours after transfection, the cells were treated with MG132 (10 μM) for 7 h, lysed on ice with lysis buffer (6 M guanidinium-HCl, 10 mM Tris-HCl pH 8.0, 0.1 M Na_2_HPO4/NaH_2_PO4, 5 mM imidazole, and 10 mM β-mercaptoethanol) for 30 min and boiled. The total cellular lysates were centrifuged and immunoprecipitated with an anti-Myc antibody overnight at 4 °C. Then, western blotting was conducted with an anti-HA antibody. For the *in vitro* ubiquitination assay, the lysates were incubated with Ni-NTA beads (Life Technologies) at room temperature for 3 h. Then, the pulled down proteins were washed once with buffer A (8 M urea, 0.1 M Na2HPO4/NaH2PO4, 10 mM Tris-HCl pH 8.0 and 10 mM β-mercaptoethanol) and once with buffer B (8 M urea, 10 mM Tris-HCl pH 6.3, 0.1 M Na2HPO4/NaH2PO4 and 10 mM β-mercaptoethanol) with 0.2% Triton X-100 and once with Buffer B with 0.1% Triton X-100. The ubiquitinated proteins were eluted by boiling in 5x SDS sample buffer and subjected to 10% SDS‒PAGE analysis with specific antibodies.

### Protein assay methods

For western blotting analysis, cells were collected after different treatments and lysed in RIPA buffer (Beyotime Biotechnologies) on ice for 30 min. Total protein was separated on a 10% SDS‒PAGE gel (Epizyme Biotechnolgia) and transferred to a PVDF membrane. The membrane was blocked with a Western Rapid Kit (GeneScript) at room temperature for 15 min. Then, the membrane was incubated with specific primary antibodies diluted from the Western Rapid Kit and incubated overnight at 4 °C. Next, the membranes were washed with TBS-T three times and incubated with the corresponding secondary antibodies at room temperature for 1 h. An enhanced chemiluminescence (ECL) kit (Beyotime Technologies) was used to visualize the results. Densitometric analysis of Western blot results using ImageJ (v1.53) software (NIH).

For coimmunoprecipitation (co-IP) analysis, the cells were lysed *via* co-IP lysis buffer (Beyotime Technologies) supplemented with protease inhibitors and PhosSTOP (Roche). Immunoprecipitation of protein was conducted using 1 μl of specified antibodies that were bound to Sepharose G beads (Abcam) (IgG was used as the negative control). The protein was eluted under reducing–denaturing conditions in 5x SDS sample buffer at 95 °C for 5 min. Then, the proteins were subjected to immunoblotting analysis.

### Measurement of protein half-life

Before the different treatments, CHX was added to the culture medium at a final concentration of 50 μg/ml. Then, the cells were subjected to various treatments and harvested at the indicated time points. Then, the total cells were harvested and lysed on ice for 30 min. The total proteins were subjected to 10% SDS‒PAGE and transferred to PVDF membranes. The target proteins were quantified *via* ImageJ software (ver. 1.61; NIH). The quantified proteins were normalized relative to the loading control.

### GST pull-down and *in vitro* phosphorylation assays

For GST, the DNA fragments of YAP encoding amino acids 293--490 of wild-type YAP and of YAP-Mut encoding amino acids 293--490 of YAP with S338A/T412A mutations were subcloned from vectors carrying Myc-YAP or Myc-YAP (S338A/T412A), respectively. These DNA fragments were inserted into the pGEK-2TK plasmid (Beyotime) to generate GST fusion proteins. These vectors were then transformed into BL21(DE3) competent *E. coli* cells (Beyotime Biotechnologies). The transformed bacteria were grown overnight and incubated with 1 mM IPTG at 25 °C for 24 h. Then, the proteins were purified *via* glutathione-Sepharose 4B (Life Technologies) according to the manufacturer's instructions. The eluted proteins were desalted *via* PD-10 Sepharose G-25 supplemented with 10% glycerol (Life Technologies) according to the manufacturer's instructions. For *in vitro* phosphorylation assays, exogenous Flag- GSK-3β was immunoprecipitated from cells by antibodies against Flag, and then immunoprecipitates were incubated with recombinant GST-YAP (WT or Mut) and [γS] ATP in kinase buffer (Cell signaling Technology) according to the manufacturer's instructions. The samples were subjected to 10% SDS‒PAGE analysis.

### Quantitative real-time PCR (qRT‒PCR)

After different treatments, total RNA was extracted from cells *via* TRIzol reagent (Life Technologies) following the manufacturer's instructions. DNA was removed by the addition of DNase I (Beyotime Biotechnologies) at 37 °C for 20 min. The reaction was stopped by the addition of Stop solution and incubation at 70 °C for 10 min cDNA was synthesized *via* a High-Capacity cDNA Reverse Transcription Kit (Qiagen). Quantitative PCR was performed *via* SYBR Supermix (Takara) on a QuantStudio Real-Time PCR system (Applied Biosystems). GAPDH mRNA levels were used as the internal control. The following primers were used in the study: YAP, (F): 5′-CAAATCCCACTCCCGACA-3′; (R): 5′-TCTGACCAGAAG ATG TCT TTG C-3′. GAPDH (F): 5′-GGAGCGAGATCCCTCCAAAAT-3′; (R): 5′-GGCTGTTGTCATACTTCTCATGG-3′.

### Cell viability assay

Cell viability was assayed with the aid of a Cell Counting Kit-8 (CCK-8) proliferation assay kit (Dojindo) in accordance with the manufacturer's instructions. Briefly, 96-well plates were seeded at a density of 5 × 103 cells/well. The cells were then subjected to different treatments for varying durations. Next, each well received 10 μl of CCK-8 solution, which was maintained at 37 °C for an additional 2 h. The results were read at 450 nm by a microplate reader (BioTek), and the process was repeated three times.

### Immunofluorescence

T-24 and 5637 cells were transfected with YAP and FBXO9 expression plasmids and subsequently fixed with 4% paraformaldehyde. Then, the fixed cells were incubated with primary antibodies overnight at 4 °C. Nuclei were stained with 1 μM 4′,6-diamidino-2-phenylindole (DAPI) for 10 min. Fluorescence images were captured *via* a confocal laser LSM 5 Pa microscope (Carl Zeiss).

### Statistical analysis

Prism 9.0 software (TreeStar) was used for the statistical analyses. The findings are presented as the means ± SDs. For between-group comparisons, Student's *t* test (two-tailed distribution, two-sample and unequal variance) was employed, and comparisons among multiple groups were performed *via* one-way analysis of variance (ANOVA), followed by Tukey's *post hoc* test. *p* < 0.05 (two-tailed) indicated statistical significance.

## Data availability

Data available upon reasonable request.

## Supporting information

This article contains supporting information.

## Conflict of interest

The authors declare that they have no conflicts of interest with the contents of this article.

## Figures and Tables

**Figure S2:**