# Supplementary material for: FBXO9 mediated the ubiquitination and degradation of YAP in a GSK-3β-dependent manner
Source: J Biol Chem. 2025 Sep 1;301(10):110652. doi: 10.1016/j.jbc.2025.110652 (PMC12509758; doi:10.1016/j.jbc.2025.110652)
Supplement: Table S1 [file mmc1.docx]

Table 1. List of siRNA and sgRNA sequences

| Target | siRNA Sequence 5’-3’ |
| --- | --- |
| Ctrl (NC) | GCAAACCAGCACCUUAGCUCC |
| Cul1 #1 | GCUCUACACUCAUGUUUAU |
| Cul1 #2 | AACCAAGGACUUAAUGUAUU |
| Cul2 #1 | GCCCUUACGUCAGUUGUAAAUUACA |
| Cul2 #2 | AA GCU UCU ACG GUU AUC AUU |
| Cul3 #1 | GAGUGUAUGAGUUCCUAUU |
| Cul3 #2 | AA CCU AAU GAG CUA CCA AUU |
| Cul4A #1 | GAACUUCCGAGACAGACCU |
| Cul4A #2 | AA GCU GGU AUU GCU CAU AUU |
| Cul4B #1 | CACCGUCUCUAGCUUUGCUAA |
| Cul4B #2 | AA CUA GGU GUAAUUGCCAUU |
| Cul5 #1 | CUGGAGGACUUGAUACCGGAA |
| Cul5 #2 | AAUUAGUAGCUAUCAGUAUU |
| FBXO9 #1 | GAAGCCAUCAAGUUUAUCGUA |
| FBXO9 #2 | GAGGUCCUGAUGUACAUCUUCCGAU |
| GSK-3β #1 | GGCCACAAGAAGUCAGCUAUAC |
| GSK-3β #2 | GUUGAUCUUUGGAGCCACUG |
|  |  |
| Target | sgRNA sequence |
| Ctrl | GCCACCUUACCAUCAUCA |
| FBXO9 | CCCCCCTCAGGCAGAAGCTG |
